# Detecting Dairy Cattle Protective Behaviors via a Multi-Stage Attention SlowFast Network

**DOI:** 10.3390/ani16091321

**Published:** 2026-04-26

**Authors:** Bo Zhang, Jia Li, Feilong Kang, Yongan Zhang, Yu Xia, Yanqiu Liu, Jian Zhao

**Affiliations:** 1 College of Computer and Information Engineering, Inner Mongolia Agricultural University, Hohhot 010018, China; zhguzibo@163.com (B.Z.); zhangya@imau.edu.cn (Y.Z.); yuxia@imau.edu.cn (Y.X.); liuyq@imau.edu.cn (Y.L.); zhaojian@emails.imau.edu.cn (J.Z.); 2 Inner Mongolia Autonomous Region Key Laboratory of Big Data Research and Application of Agriculture and Animal Husbandry, Hohhot 010018, China; 3 College of Mechanical and Electrical Engineering, Inner Mongolia Agricultural University, Hohhot 010018, China; kfl@imau.edu.cn

**Keywords:** dairy cattle welfare, protective behavior detection, computer vision, SlowFast, multi-stage attention mechanism

## Abstract

Protective behavior in dairy cattle is one of the important potential indicators of their health and welfare status, and the precise detection of this behavior is of great significance for improving pasture management. However, existing methods face challenges, including capturing rapid motions, excessive background interference, and sample imbalance in complex agricultural environments. In response to these challenges, we proposed a Multi-Stage Attention SlowFast (MSA-SlowFast) model based on the improved SlowFast network to explore the model’s ability to distinguish between normal and protective behavior of dairy cattle. It achieves performance improvement through three core modules: the Multi-Path Balanced Head (MPBHead) for alleviating category imbalance, the Spatio-Temporal Convolutional Block Attention Module (ST-CBAM) for enhancing key feature extraction, and the 7 (BAF) for promoting multi-path feature complementarity. Additionally, we proposed novel timing-aware oversampling methods and dynamic loss adjustment mechanisms to further improve the detection performance of minority-class protective behaviors. Finally, a spatio-temporal-oriented dairy cattle protective behaviors dataset is constructed. Experimental results demonstrate that the proposed MSA-SlowFast model achieves 79.41% mAP, surpassing the standard SlowFast (70.58%) and Slow-only (68.21%). Further validation shows that the model exhibits high detection confidence in four specific actions labeled as protective behavior: 0.97 for tail swaying, 0.90 for head shaking, 0.92 for ear flapping, and 0.90 for leg kicking. These preliminary results show that the method proposed in this study has certain feasibility and reference value for the detection of protective behavior of dairy cattle under our constructed dataset.

## Simple Summary

This study focuses on the automatic detection of protective behavior, a key health indicator of dairy cattle. However, this task is challenged by rapid movement, background interference, and sample imbalance in complex farm environments. Therefore, we propose a Multi-Stage Attention SlowFast (MSA-SlowFast) model based on the improved SlowFast network. It employs three key modules: Multi Path Balanced Head (MPBHead) to address category imbalance, Spatio-Temporal Convolutional Block Attention Module (ST-CBAM) to improve key feature extraction, and Bidirectional Adaptive Fusion Module (BAF) to foster feature complementarity across multiple paths. At the same time, this study also proposes an innovative timing-aware oversampling method and dynamic loss adjustment mechanism to further improve the detection performance of minority protective behaviors. Experimental results demonstrate that the proposed MSA-SlowFast model achieves 79.41% mAP, surpassing the standard SlowFast (70.58%) and Slow-only (68.21%). Further validation shows that the model exhibits high detection confidence in four specific actions labeled as protective behavior: 0.97 for tail swaying, 0.90 for head shaking, 0.92 for ear flapping, and 0.90 for leg kicking.

## 1. Introduction

In recent years, China’s dairy cattle breeding industry has developed steadily, with both the number of cattle in stock and milk production continuously increasing. According to data from the National Bureau of Statistics, in 2024, the number of cows in stock in China reached 100.465 million, marking a 5.1% increase compared to 2020. Milk production amounted to 40.794 million tons, representing a 16.6% increase from 2020 [1]. While the industry is developing rapidly, in order to ensure the welfare of dairy cattle, cattle behavior detection technology has gradually become an important development direction in the dairy farming industry [2]. The behavior patterns of dairy cattle are closely related to their health status. Regular feeding, rumination, and resting behaviors are important manifestations of cattle health, while abnormal changes in behavior are often early signals of disease, stress, or environmental discomfort [3]. Among various abnormal behaviors, protective behavior, as an adaptive response of cattle to external stimuli, is often manifested through actions such as tail-swaying, head-shaking, ear-flapping, and leg-kicking to repel mosquitoes or alleviate discomfort caused by other external stimuli [4]. Specifically, due to mosquitoes, dairy cattle will frequently sway their tails and kick their legs, which is a direct source of stress for cows and may lead to a decrease in subsequent milk production [4,5,6]. Importantly, head-shaking and ear-flapping are recognized as core pain-related behaviors in standardized assessments, with their frequency demonstrably increasing during painful procedures such as cautery disbudding and decreasing with effective analgesia [7]. This type of protective behavior is essentially a passive response of dairy cattle due to discomfort or pain, and is a key indicator reflecting their health and welfare status. Therefore, from a welfare perspective, by monitoring the protective behavior of dairy cattle, potential health problems can be detected in a timely manner, and corresponding intervention measures can be taken to improve the welfare of cattle and the economic benefits of pastures [8].

In recent years, significant progress has been made in animal behavior recognition methods based on deep learning. These methods are mainly divided into two categories: pose estimation-based methods and non-pose estimation-based methods. The pose estimation-based methods mainly detect key points of the animal’s body, construct a pose skeleton model, and then combine machine learning algorithms to capture the animal’s behavior. Classic pose estimation-based methods include DeepLabCut [9], SLEAP [10], HRNet [11], etc. Russello et al. [12] used T-LEAP pose estimation to track nine keypoints of dairy cows and computed six locomotion traits, achieving lameness classification accuracy of 80.1% by combining all traits—a notable improvement over 76.6% using a single trait. Fang et al. [13] extracted six features and combined them with Naive Bayes to accurately classify the daily behavior of broiler chickens. The classification accuracy of resting behavior reached 96.23%, while walking and running had lower classification accuracy due to similar motion amplitudes. Lin et al. [14] used HRNet to detect bird keypoints, extract bird features for accurate bird pose recognition, and combined ResNet18 for behavior classification. The results showed that the average accuracy in classifying eight bird behaviors can reach 87.99%. Although the above research has achieved certain results, there is a common problem with such methods, which is that their performance is highly dependent on the accuracy of pose estimation. In complex farming environments, there are many unfavorable factors, such as occlusion between animals and drastic changes in lighting, which can pose significant challenges to the stable detection of key points and lead to incorrect behavior recognition results.

End-to-end non-pose estimation-based methods can be used to overcome the above limitations. This method can automatically extract behavior-related features directly from raw video data, without the need to detect key points on the animal’s body, and can directly locate the animal’s behavior. Classic methods include Two Stream CNN [15], I3D [16], SlowFast [17], ResNet [18] +LSTM [19], etc. Hao et al. [20] proposed a TSML model based on Two Stream CNN, which utilizes collaborative learning student networks and weighted fusion of RGB and stream data branches to improve the accuracy of pig behavior recognition to 96.52%. However, it relies on optical flow input and has high computational overhead, which promotes the exploration of single-stream 3D convolution methods. The E3D method proposed by Wang et al. [21] combines 3D convolution with depthwise separable convolution and introduces an efficient channel attention mechanism to accurately and quickly identify the basic movement behavior of cattle, with an accuracy improvement of 4.06% compared to I3D. Liu et al. [22] integrated the SE attention module into the SlowFast slow path and introduced a new loss function to solve class imbalance, achieving an accuracy of 93.56% and 98.77% in recognizing horse sleep and eating behavior, respectively. The AdRes3D BiLSTM model developed by Yuan et al. [23] integrates multiple mechanisms to efficiently recognize sheep behavior in real-time. While maintaining a high accuracy of 98.72%, it compresses the memory to 28.03 MB and achieves a frame rate of 52.79 f/s, achieving a win–win situation of accuracy and efficiency. In addition to these video classification architectures, object detection-based approaches, particularly those leveraging YOLO (You Only Look Once) networks [24], have emerged as a powerful and efficient paradigm for directly localizing and identifying specific behaviors in real-time. This is exemplified by recent work such as that of Smith et al. [25], who developed a YOLO-based system capable of accurately detecting dairy cattle and classifying key behaviors like feeding directly from video streams, demonstrating the practical advantages of this approach for on-farm monitoring applications.

Despite these improved architectures (e.g., SlowFast with SE attention [22], EML-SlowFast with Efficient Channel Attention and Large Kernel modules [26], and SCTS-SlowFast with Temporal-Spatial attention [27]) have achieved some success, detecting dairy cattle protective behaviors in practical farm scenarios still face some complex challenges that existing variants have not fully addressed. These issues can be summarized as follows: (1) The protective behavior of cows is usually short-term and rapid, which makes it difficult for traditional static image analysis methods to effectively capture [28]. (2) Most existing behavior detection methods rely on large-scale annotated datasets, and in the field of cow protective behavior, publicly available dataset resources are scarce [29]. (3) In behavior data, there exists a class imbalance issue between normal behavior and protective behavior, which may adversely affect the model’s detection accuracy [30].

To this end, the objective of this study is to develop and evaluate a lightweight Multi-Stage Attention SlowFast (MSA-SlowFast) model framework for detecting protective behavior of cattle in farm settings. In this study, a video dataset of cow behavior was made, and two kinds of behavior categories, normal behavior and protective behavior, were annotated. In order to alleviate the problem of class imbalance in data, a time series-aware oversampling enhancement strategy is proposed. In addition, by improving the loss function and classification header structure, the sensitivity of the model to minority protective behavior recognition is enhanced.

## 2. Materials and Methods

### 2.1. Dataset

#### 2.1.1. Data Collection

The cow behavior video data used in this study were collected from three large-scale pastures near Hohhot, Inner Mongolia, China. Video recordings were captured from June to September 2025, during two daily time windows: 9:00–11:00 and 14:00–16:00. These time windows were selected because they correspond to the peak daily activity periods for dairy cows, during which protective behaviors are more frequently observed due to increased mosquito activity and higher ambient temperatures. This study used a DJI Pocket 3 camera, chosen for its portability and real-time preview capability, which allows on-site filtering and prioritized capture of protective behavior segments to enrich the dataset. The camera was positioned at a fixed location 2–3 m outside the cowshed with a stable overhead squint angle (approximately 45° to the ground). This angle can systematically cover the main areas of feeding, rest, and activity of cows, while minimizing mutual shielding. During the data collection phase, approximately 100 Holstein cows were filmed. A total of 93 video segments were captured, with each segment lasting 10–15 min, amounting to approximately 1200 min of footage. The resolution of the captured video data was 1280×720 pixels, with a frame rate of 30.0 frames per second.

#### 2.1.2. Dataset Construction

Our dataset is a composite one, formed by integrating self-collected video segments with behavior videos from the publicly available MultiCamCows2024 dataset. The Multicamcows2024 contains multi-view recordings from commercial farms, including normal behaviors such as walking, lying down, and standing [31]. This integration enriches data diversity and enhances the model’s generalization across varied real-world conditions.

In order to construct a precise and representative dataset of protective behavior, we rigorously screened the videos based on the following criteria: (1) At least 80% of the whole body of the target cow is visible. (2) No obstruction from other cows or facilities lasting for more than 1 s. (3) The duration of the fragment shall not be less than 5 s to ensure that it contains a complete sequence of behaviors. For the multi-view videos in the MultiCamCows2024 dataset, we selected the single view that was closest to the shooting angle of the self-collected data for alignment to ensure the consistency of the viewpoints throughout the dataset. Based on these criteria, we compiled a video subset representing the original protective behavior data, comprising a total of 110 video segments. Each video clip should be controlled within 8 to 10 s.

**Figure 1:**
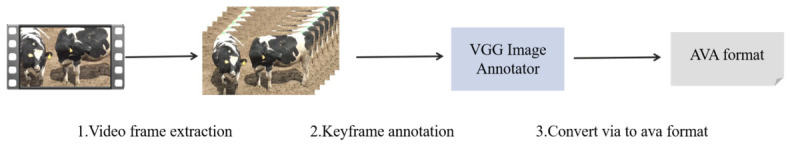
Dataset production process. VGG Image Annotator (VIA) is an open-source manual annotation tool developed by the Visual Geometry Group (VGG) at the University of Oxford.

To construct a dataset for model training, this study implemented a systematic video data annotation process, as shown in Figure 1. The dataset used in the study was annotated according to the publicly available behavior detection AVA dataset format [32]. This format was chosen because its frame-level, multi-label annotation structure is well-suited for capturing transient and co-occurring protective behaviors (e.g., tail-swaying with concurrent head-shaking), and its widespread adoption ensures compatibility with existing behavior recognition pipelines.

**Figure 2:**
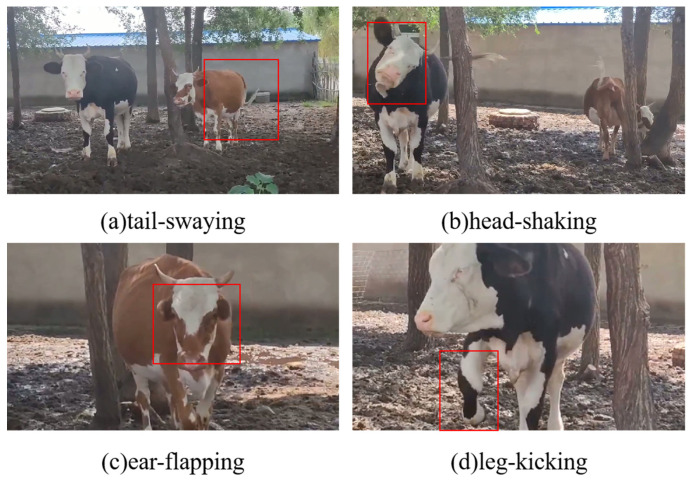
Schematic diagram of different protective behaviors of dairy cattle.

In accordance with the AVA dataset format requirements, two methods were used for frame extraction from the video data: extracting 1 frame per second and extracting 30 frames per second. A total of 30,808 frames were extracted. Among these, frames extracted at a rate of 1 frame per second were labeled. In the process of annotation, we annotated the behavior category (normal behavior or protective behavior) of the dairy cattle in the video and its spatio-temporal position bounding box in the image using the VGG Image Annotator (VIA) software (version 2.0.11). Table 1 details four actions that can be defined as protective behaviors: tail-swaying, head-shaking, ear-flapping, and leg-kicking. These actions were collectively categorized as protective behavior, which is strictly mutually exclusive from normal behavior. Figure 2 provides a schematic diagram of frames annotated as protective behavior. Following the annotation phase, the preliminary results were exported in CSV format. To enable compatibility with SlowFast, the data structure was then converted to conform to the AVA v2.2 dataset specification. A total of 2152 labeled images were obtained during this stage, including 1897 images of normal behavior and 255 images of abnormal behavior.

The dataset was divided at the video level into a training set and a test set in an 8:2 ratio, resulting in 88 videos for training and 22 videos for testing. The training set contained 24,417 frames, from which 1686 keyframes were selected and annotated. The test set contained 6391 frames, with 466 keyframes annotated.

#### 2.1.3. Dataset Augmentation

To enhance model robustness, data augmentation was applied during data loading, which increased the amount of training data and alleviated the problem of data scarcity. This process increased the dataset’s size and diversity. However, it also revealed a more challenging problem: a class imbalance between a small amount of protective behavior and a large amount of normal behavior data. During model training, the data loader typically employs uniform random sampling [33]. Due to this strategy, the rare behavior categories are sampled far less frequently per epoch than the majority categories, which hinders the model’s ability to learn their discriminative features. Therefore, merely increasing data diversity is insufficient; it is also necessary to augment the sample size of rare categories in a targeted manner.

**Figure 3:**
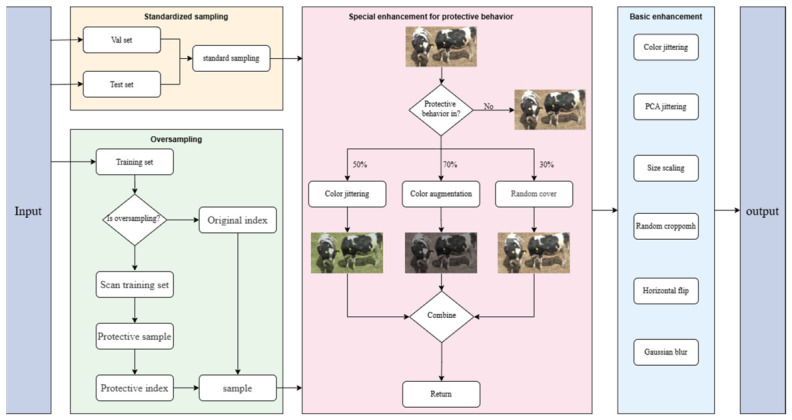
Data augmentation process.

As shown in Figure 3, oversampling was applied only to the training set, while the validation and test sets kept the standard sampling strategy. The first stage of oversampling constructed a sample pool of protective behavior by precomputing video frame indices. During training, whether to trigger oversampling was determined before each iteration: generate a [0, 1) random number r, which is compared with the preset probability p. If r < p, oversampling was triggered, and samples were randomly selected from the sample pool to replace the original indices of the current batch, while ensuring the continuity of video timing. After expanding the sample size, the process entered a specialized enhancement phase for protective behavior. In this phase, all images labeled as protective behavior were first identified for specialized enhancement. This enhancement employed three methods with the following activation probabilities: 50% for color jitter, 70% for color enhancement, and 30% for random occlusion. Finally, all data are processed through a unified preprocessing process, including basic enhancement operations such as color jittering, PCA jittering, and size scaling. The final output includes: the multi-path tensor of the video clip, the corresponding bounding box and behavior label, as well as metadata such as video index and timestamp.

### 2.2. Method

#### 2.2.1. Establishment of SlowFast Module

**Figure 4:**
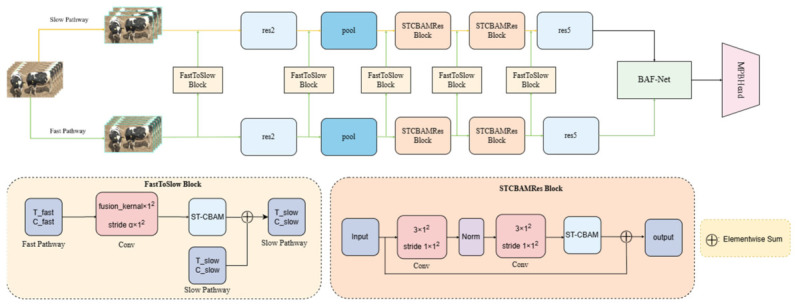
MSA-SlowFast model structure.

Given that protective behavior in dairy cattle involves both slow postural changes and rapid motion patterns, we adopted the SlowFast architecture as the backbone network. As illustrated in Figure 4, the proposed Multi-Stage Attention SlowFast network (MSA-SlowFast) extends this dual-pathway design with multi-stage attention mechanisms specifically tailored for detecting protective behavior in dairy cattle.

The SlowFast network comprises two pathways: a Slow pathway and a Fast pathway. The Slow pathway primarily extracts spatial semantic information that captures the sustained postural changes characteristic of behaviors such as leg-kicking. Conversely, the Fast pathway employs a high frame rate 3D CNN with fewer channels and narrower convolution width to encode rapid motion details, which are essential for identifying swift actions like tail-swaying and head-shaking. Both pathways adopt the network structure of 3D ResNet. Features from both pathways are fused through lateral connections, enabling the model to integrate complementary temporal cues.

The SlowFast network is parameterized by τ, α, and β, where τ denotes the temporal sampling stride, α the frame rate ratio between the two pathways, and β their channel width ratio. Specifically, the frame rate of the Fast path is α times that of the Slow path (α>1), and the channel width of the Fast path is β times that of the Slow path (β<1).

Based on the SlowFast framework, three core hyperparameters were empirically optimized on our dataset for protective behavior detection: the temporal sampling stride τ was set to 8, the frame rate ratio α was set to 4, the channel width ratio β was set to 1/8. These values were selected following preliminary experiments in which they demonstrated superior performance.

Compared to SlowFast, the proposed MSA-SlowFast integrates Spatio-Temporal Convolutional Block Attention Module (ST-CBAM) at the feature fusion connection point of the FastToSlow system, enabling the feature fusion process from the Fast-to-Slow path to adaptively focus on important spatio-temporal regions. Furthermore, the original Res3 and Res4 blocks are replaced with STCBAMRes blocks, thereby strengthening the expressiveness of mid-level features. To further refine the architecture, a dedicated Bidirectional Adaptive Fusion Module (BAF) is added prior to the detection head, which is itself substituted with a Multi-Path Balanced Head (MPBHead) for robust multi-scale detection.

The FastToSlow Block is designed to fuse features of the Fast pathway into the Slow pathway. Specifically, the temporal length T_fast and channel dimension C_fast of the Fast pathway are determined by hyperparameters α and β, such that T_fast =α×T_slow and C_fast =C_slow//β, where T_slow and C_slow denote the temporal length and channel dimension of the Slow pathway, respectively. Before fusion, a 3D convolution operation with a kernel size of [fusion-kernel, 1, 1] and a stride of [α,1, 1] is applied. This operation not only reduces temporal length from T_fast to T_slow, but also compresses the channel dimension by a predefined compression ratio r (set to 0.5), yielding C_fast×r channels. Subsequently, the adapted features are processed by the integrated ST-CBAM module to recalibrate their spatio-temporal importance through adaptive weighting. Finally, these weighted features are fused with the original Slow pathway features via element-wise addition.

#### 2.2.2. MPBHead Module

**Figure 5:**
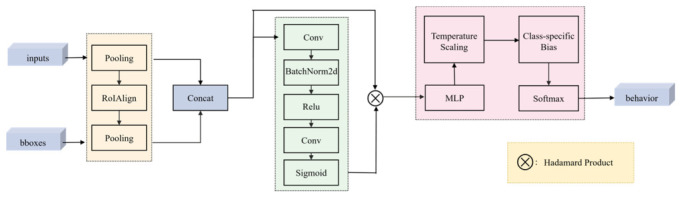
MPBHead model structure. Input represents the spatio-temporal features of the Slow Pathway and Fast Pathway, bbboxes represents the bounding box input, and behavior represents the probability distribution of behavior.

While the native detection head of the SlowFast network can effectively fuse dual-path features, it lacks sensitivity to class-imbalanced datasets, as it is primarily designed for general action recognition. With normal behavior samples far exceeding protective behavior samples, the standard head tends to bias predictions toward the majority class, increasing the risk of missing rare but critical protective actions. To address this limitation, we designed the Multi-Path Balanced Head (MPBHead), a task-specific detection head tailored for imbalanced bovine behavior recognition. As illustrated in Figure 5, the MPBHead consists of three core components: feature fusion, attention weighting, and dual balanced classification.

The feature fusion stage takes the feature maps from the Slow and Fast pathways as input, with channel dimensions C_slow and C_fast, respectively. Firstly, temporal average pooling is applied to both feature maps, reducing their temporal length to 1. Subsequently, for a total of N dairy cattle targets to be classified in this batch of inputs (specified by the input parameter bboxes), ROIAlign is used to extract corresponding region features from both pooled feature maps. After spatial pooling, the resulting region features are concatenated into a unified feature matrix of dimension [N, C_slow+C_fast], which serves as input for subsequent attention weighting modules.

Following feature fusion, a lightweight channel attention module is introduced to adaptively recalibrate the importance of feature channels. This module is constructed as a bottleneck comprising two sequential 1×1 convolutional layers. The first layer compresses the concatenated feature channels from C_total (C_total = C_slow + C_fast) to C_total//4, which is then processed by Batch Normalization and a ReLU activation function for non-linear transformation and stable training. The second layer subsequently expands the channel dimension back to the original C_total. A Sigmoid activation function is applied to generate a channel-wise attention weight vector, with each element constrained to the range [0, 1]. This weight vector is then multiplied element-wise with the initial concatenated feature map, producing a recalibrated feature representation that amplifies channels critical for distinguishing protective behaviors.

The refined features are subsequently flattened and fed into a dual-balanced classification module, specifically designed to address the severe class imbalance between normal and rare protective behaviors. This stage is implemented through a Multi-Layer Perceptron (MLP) classifier followed by a strategically calibrated softmax activation. The MLP classifier is a two-layer fully connected network. The first layer projects the C_total-dimensional input into a hidden representation of dimension hidden_dim (set to 512), followed by Batch Normalization, a ReLU activation, and a Dropout layer for regularization. A second Dropout layer precedes the final linear layer, which outputs the raw logits for all num_classes. To ensure balanced optimization, the logits z are calibrated before the final activation using two key mechanisms: temperature scaling and learnable class bias. The calibration follows Equation (1).

(1) $$z^{\prime }=\frac{z}{T}+b,$$

In Equation (1), T (where T>0) is a temperature hyperparameter that softens the output probability distribution, mitigating overconfidence in the majority class. The vector b is a set of learnable class bias parameters. It explicitly compensates for protective behavior categories with sparse samples by directly shifting the decision boundary of the classifier, thereby actively alleviating the problem of class imbalance at the output level.

Finally, the calibrated logits $z^{\prime }$ are passed through a Softmax function to obtain the final class probability distribution.

#### 2.2.3. ST-CBAM Module

ST-CBAM introduces a temporal dimension and spatio-temporal joint attention on the basis of traditional CBAM [34], specifically designed for processing three-dimensional spatio-temporal data such as video sequences. This architectural design is particularly well-suited for protective behavior detection in dairy cattle, as these behaviors are inherently defined by coordinated spatial and temporal patterns. Consequently, accurate recognition requires the model to simultaneously attend to the spatial locations where actions occur and their temporal evolution over time.

**Figure 6:**
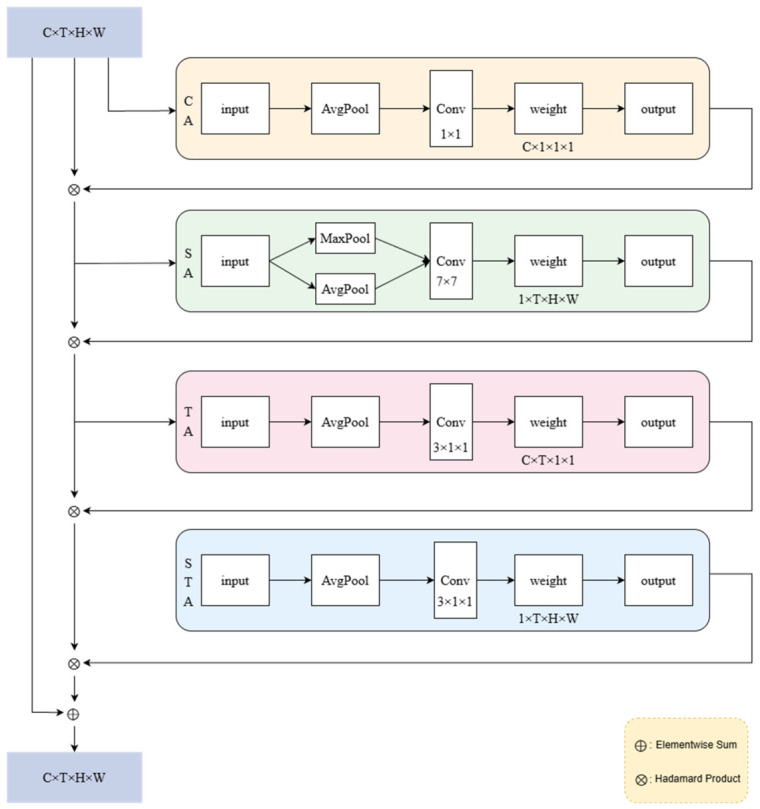
ST-CBAM model structure.

As illustrated in Figure 6, the module comprises four sequentially connected attention sub-modules: channel attention, spatial attention, temporal attention, and spatio-temporal joint attention. Each sub-module refines features along a specific dimension, and all outputs are integrated with the original input via residual connections. The module takes a feature tensor $X\in R^{B\times C\times T\times H\times W}$, where *B* is the batch size, *C* is the number of channels, *T* is the time step, and *H* and *W* are the spatial height and width, respectively.

The channel attention adaptively recalibrates feature channels to emphasize those most relevant to protective behaviors. Obtain channel statistical information through global average pooling, construct a bottleneck structure using two convolutions of 1×1×1 to learn inter-channel dependencies, and finally generate channel weights $A_{c}$ through the Sigmoid function. The weight is multiplied by the input feature *X* channel by channel to obtain $Y_{c}$, which enhances the important channel and suppresses the secondary channel, improving the feature representation ability, as shown in Equation (2).

(2) $$Y_{c}=X\cdot A_{c},$$

The spatial attention highlights body regions where protective actions typically occur (e.g., the tail, head, and legs). Obtain spatial features through channel dimension mean pooling and maximum pooling, concatenate the two, and use 7×7 spatial convolution to learn spatial importance distribution, and generate spatial weights $A_{s}$ through Sigmoid. This weight is multiplied by the feature map $Y_{c}$ position by position to obtain $Y_{s}$, highlighting key spatial regions, suppressing background noise, and improving the spatial perception ability of the model, as shown in Equation (3).

(3) $$Y_{s}=Y_{c}\cdot A_{s},$$

The temporal attention focuses on key motion phases within a behavior sequence. Compressing spatial information through spatial dimension pooling, capturing local temporal context using two 3×1×1 temporal convolutions, and generating temporal weight $A_{t}$ through Sigmoid. This weight is multiplied with the feature $Y_{s}$ step by step to obtain $Y_{t}$, which enhances keyframe features, suppresses redundant temporal information, and improves the model’s perception of dynamic changes, as shown in Equation (4).

(4) $$Y_{t}=Y_{s}\cdot A_{t},$$

The spatio-temporal joint attention integrates these cues to capture the complete action pattern. Compress the channel dimension to 1 through two 3×1×1 convolutions while maintaining temporal processing capability, and generate spatio-temporal weights $A_{st}$ through Sigmoid. The weight is multiplied by the feature $Y_{t}$ at each spatio-temporal position and the input feature *X* to obtain the final output *Y*, achieving spatio-temporal collaborative optimization, enhancing key spatio-temporal region features, and improving the modeling ability of the model for complex spatio-temporal patterns, as shown in Equation (5).

(5) $$Y=X+Y_{t}\cdot A_{st},$$

#### 2.2.4. BAF Module

**Figure 7:**
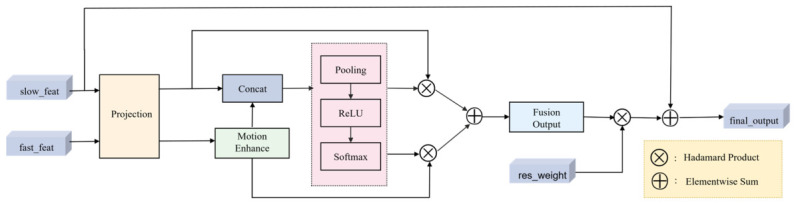
BAF model structure. slow_feat and fast_feat represent the feature representations of the Slow pathway and Fast pathway, respectively. res_weight is the weighted weight, and final_output is the final fused output result.

In the original SlowFast network, feature fusion relies on simple lateral connections and direct addition. While computationally efficient, this approach lacks adaptive weighting, which poses a critical limitation for detecting protective behavior in dairy cattle. These behaviors exhibit rich spatio-temporal variations: tail-swaying involves rapid oscillatory movements that require precise motion encoding, while leg-kicking combines sustained postural changes with sudden dynamic actions. Because a uniform fusion strategy cannot simultaneously accommodate such diverse demands, it fails to capture the full spectrum of protective behaviors, consequently constraining the model’s representational capacity. To address this limitation, we proposed the BAF module. The BAF module achieves efficient fusion of semantic information from the Slow pathway and motion details from the Fast pathway through three key mechanisms: bidirectional feature projection, motion feature enhancement, and spatio-temporal neighborhood fusion. The architecture of the BAF module is shown in Figure 7.

Given the Slow pathway features $F_{s}\in R^{B\times C_{s}\times T\times H\times W}$ and the Fast pathway features $F_{f}\in R^{B\times C_{f}\times T_{f}\times H_{f}\times W_{f}}$, the module first projects both sets of features into a unified dimensional space. Specifically, the Slow pathway features undergo a 1×1×1 convolution to halve the channel count, which retains high-level semantics while removing redundancy. The Fast pathway features are processed by an identical projection mechanism to align their dimensions with those of the projected Slow features. The outputs of this step are denoted as $F_{s}^{\prime }$ and $F_{f}^{\prime }$. If a spatial or temporal dimension mismatch exists, trilinear interpolation is applied to achieve size alignment. Subsequently, a 3×1×1 convolution is applied along the temporal dimension of $F_{f}^{\prime }$. This operation captures dynamic changes between consecutive frames, producing motion-enhanced features $F_{f}^{ehanced}$. Finally $F_{f}^{ehanced}$ with the projected Slow features $F_{s}^{\prime }$ to form the combined representation $F_{concat}$.

Subsequently, $F_{concat}$ passes through the motion feature enhancement mechanism. This mechanism first aggregates global feature statistics via global average pooling to capture overall behavioral patterns. Subsequently, a bottleneck structure consisting of two fully connected layers is utilized for attention weight calculation. The first fully connected layer compresses the feature dimension and introduces non-linear transformations, while the second fully connected layer outputs the attention weights of two channels. Finally, normalization is performed using the Softmax function to obtain the attention coefficients $A_{s}$ and $A_{f}$ for both Slow and Fast pathways. The attended feature representation $F_{att}$ is obtained by applying these coefficients to the original concatenated features, as formulated in Equation (6).

(6) $$F_{att}=F_{s}^{\prime }\cdot A_{s}+F_{f}^{ehanced}\cdot A_{f},$$

The motion feature enhancement output $F_{att}$ is then passed to the fusion stage, where spatio-temporal neighborhood fusion is conducted through a 3×3×3 3D convolution, yielding $F_{fusion}$. Subsequently, the fused features $F_{fusion}$ are multiplied by a residual weight $\alpha_{res\_weight}$ (initially set to 0.1) and then added to the original Slow pathway features $F_{s}$, as formulated in Equation (7).

(7) $$F_{final\_output}=F_{s}+F_{fusion}\cdot \alpha_{res\_weight},$$

#### 2.2.5. Improved Loss Function

Loss function is a key indicator to measure the difference between the predicted class labels and the true labels of the model, among which the cross-entropy loss function is widely used in various classification scenarios [35]. This study focuses on the detection of cow protective behavior, which is essentially a typical binary classification problem. In binary classification tasks, Binary Cross-Entropy Loss (BCE) is a commonly used loss function choice [36]. However, in the practical context of this study, we observed that the dataset exhibits a significant class imbalance. This characteristic hindered the BCE loss from effectively learning the features of the rare class during training, which consequently limited the model’s overall accuracy.

In response to the issue of class imbalance, Focal Loss was employed [37]. It adjusts sample weights dynamically during training, reducing the influence of well-classified majority samples and directing more attention to misclassified or rare-class samples. The formulation of Focal Loss is given in Equation (8).

(8) $$L_{F}=-\alpha {\left(1-p_{y}\right)}^{\gamma }log\left(p_{y}\right),$$

In Equation (8), $p_{y}$ is the prediction probability of the model for the target class, which in our model corresponds to normal behavior versus protective behavior. α is the balance factor used to adjust the influence between positive and negative samples. By assigning a higher weight to protective samples, α ensures that the minority class contributes meaningfully to the loss computation. γ controls the relative weighting of easy versus hard examples. By down-weighting the loss from easy examples, γ forces the model to focus on these difficult instances, improving its ability to detect nuanced protective behaviors that might otherwise be missed.

Focal Loss is sensitive to the setting of hyperparameters, and different parameter configurations may lead to significant fluctuations in model performance. In addition, excessively weakening the weight of easily classified samples may result in the loss of effective information in most class samples. To address these limitations, we proposed the Dual Focus Loss (DFL). It integrates Focal Loss for addressing class imbalance issues with Weighted Cross Entropy Loss for adjusting positive and negative sample contributions. The equation for Weighted Cross Entropy Loss is shown in Equation (9). When the Weighted Cross Entropy Loss is relatively large, it indicates that the classification confidence of the model on the current sample is low. At this time, the weight ratio of Focal Loss is automatically increased to enhance the model learning by utilizing its ability to mine difficult samples; On the contrary, when the Focal loss is large, the weight of the Weighted Cross Entropy Loss should be appropriately increased to maintain the basic classification ability. The equation for DFL is shown in Equation (10).

(9) $$L_{WCE}=-w_{y}log\left(p_{y}\right),$$

In Equation (9), $p_{y}$ is the prediction probability of the model for the target class, $w_{y}$ represents the weight of each category.

(10) $$L_{DFL}=focal\_weight\times L_{F}+\left(1-focal\_weight\right)\times L_{WCE},$$

In Equation (10), *focal_weight* is not a fixed hyperparameter, but an adaptive weight dynamically computed in each training batch according to Equation (11). This adaptive mechanism is particularly valuable for protective behavior detection, where the distribution of sample difficulty can vary substantially between batches. By adjusting focal_weight batch-wise, the loss function can automatically emphasize the most challenging protective behavior samples in each training iteration. The weight is clipped to the interval [0.3, 0.7] to ensure training stability, preventing extreme values that could arise from outlier batches and disrupt the learning process.

(11) $$focal\_weight=\frac{L_{WCE}}{L_{WCE}+L_{F}},$$

### 2.3. Experimental Environment and Evaluation Indicators

All experiments were conducted on a Ubuntu 18.04 system using Python 3.9, PyTorch 2.4.1, and CUDA 12.4. The hardware configuration consisted of an NVIDIA GeForce RTX 4060 Ti GPU (16 GB VRAM) and an Intel Xeon E5-2683 v4 CPU (11 cores). Through multiple rounds of validation, the following optimal hyperparameters were determined: a batch size of 4, an initial learning rate of 3 × 10^−5^, a momentum of 0.9, a weight decay coefficient of 1 × 10^−4^, and a maximum of 100 raining epochs.

The model was evaluated using the mean Average Precision (mAP) at IoU = 0.5, which is defined by the following formula:

(12) $$P=\frac{TP}{TP+FP},$$

(13) $$R=\frac{TP}{TP+FN},$$

(14) $$IoU\left(A,B\right)=\left|\frac{A\cap B}{A\cup B}\right|,$$

(15) $$AP=\int_{0}^{1}P(R)dR,$$

(16) $$mAP=\frac{1}{N}\sum_{i=1}^{n}AP_{i},$$

Equations (12) and (13) provide the calculation formulas for *P*, *R*, and *TP*, which represent the number of samples correctly predicted as positive by the model, *FN* denotes the number of positive samples incorrectly predicted as negative, and *FP* represents the number of negative samples incorrectly predicted as positive. Equation (14) represents the Intersection Over Union (IoU) threshold calculation, where *A* denotes the ground truth bounding box and *B* represents the predicted detection box. Equation (15) is the calculation formula for *AP*, where *R* is recall and *P* is precision. Equation (16) represents the average value of all categories *AP*, where *N* is the number of categories. In this study (*N* = 2), *mAP* is simplified as the average *AP* value of two categories: normal behaviors and protective behaviors.

## 3. Results

### 3.1. Identification Results of Protective Behavior

To evaluate the proposed MSA-SlowFast model, a comparative analysis was conducted under consistent experimental conditions among three key architectures: the single path Slow only network, the standard SlowFast network, and the proposed MSA-SlowFast network. As shown in Table 2, the proposed MSA-SlowFast model with a ResNet-101 backbone achieved a top mAP of 79.41%, outperforming both the Slow-only model (68.21% with ResNet-50) and the baseline SlowFast model (70.58%). Notably, even a more efficient MSA-SlowFast variant using a ResNet-50 backbone attained a mAP of 76.21%, still exceeding both baseline models.

The performance difference indicates that the deeper ResNet-101 backbone network has brought significant performance improvements to the model. Compared to the ResNet-50 version, the ResNet-101 version achieved a 3.2 percentage point improvement in mAP (from 76.21% to 79.41%). This improvement can be attributed to the stronger feature extraction ability of ResNet-101, whose deeper network structure can learn richer and more discriminative hierarchical features, which are crucial for capturing subtle, fast, and varied protective behaviors of dairy cattle.

**Figure 8:**
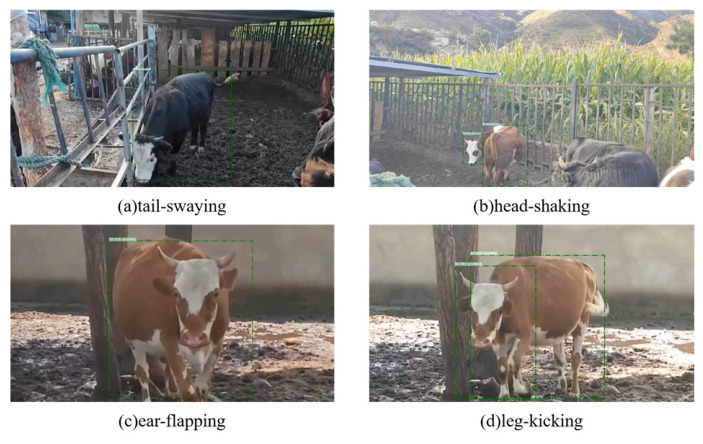
Experimental results of protective behavior. (**a**) shows the recognition results of the video containing the tail-swaying, (**b**) shows the recognition results of the video containing the head-shaking, (**c**) shows the recognition results of the video containing the ear-flapping, and (**d**) shows the recognition results of the video containing the leg-kicking.

After determining the overall effectiveness of the model, further analysis was conducted on its performance in daily cattle protective behavior videos containing four specific actions. As shown in Figure 8, the model achieved high detection confidence across all actions: 0.97 for tail swaying, 0.90 for head shaking, 0.92 for ear flapping, and 0.90 for leg kicking. The above four specific actions are marked as protection when the result is output. All confidence scores exceed 0.89, confirming the model’s robust capability in identifying dairy cattle protective behavior. Notably, tail swaying received the highest confidence, while the other three actions exhibited slightly lower but still strong scores.

Our analysis indicates that the tail swaying action possesses distinct motion characteristics that give it a detection advantage over the other three behaviors. Specifically, tail swaying exhibits a large amplitude and a clear, well-defined trajectory, making it a prominent visual cue within the video sequence. In contrast, head shaking and ear flapping are subtle, localized motions confined to the dairy cattle’s head region; their small amplitudes make them susceptible to being overlooked amidst complex backgrounds or under low resolution. Although leg kicking involves a larger motion range, it is often constrained to the lower portion of the frame and prone to occlusion, which further complicates its detection.

### 3.2. Comparison of Loss Functions

To assess the impact of loss-function optimization, we conducted a comparative study using the MSA-SlowFast architecture with three different loss functions: the proposed DFL, BCE, and Focal Loss.

As shown in Table 3, it shows that the model using standard BCE achieved the lowest mAP. Adopting Focal Loss increased mAP by 3.38%, confirming the effectiveness of focusing on hard examples. However, a more substantial breakthrough came from the proposed DFL. With an optimal configuration (α = 0.8, γ = 2.0, w_y = 1.0:2.0), DFL achieved a remarkable mAP of 79.41%—nearly 10 percentage points higher than the BCE baseline.

Further analysis revealed that category weights have the most critical impact on mAP. When category weights change, adjusting category weights while ensuring that α and γ remain unchanged will result in an overall improvement in mAP. The core reason is that the dataset used in this study has a certain degree of imbalance, and the high weight of the attention category is equivalent to forcing the model to focus on this difficult-to-learn minority class. However, experiments have shown that a weight of 1:2 is actually better than 1:4, indicating that overcompensation can disrupt the gradient balance of the loss function, causing the model to focus too much on a few classes and ignore the overall feature distribution, resulting in oscillations in the optimization process and difficulty in converging to the optimal solution. In contrast, α and γ mainly adjust the attention of the model to difficult samples. Only after the category reaches a relative balance can the adjustment of its value be effective.

### 3.3. Ablation Experiments on the Model’s Performance

To evaluate the contributions of the MPBHead, ST-CBAM, and BAF modules, we conducted an ablation study on the MSA-SlowFast model. Throughout the experiments, key hyperparameters were held constant.

As shown in Table 4, all three proposed modules contribute positively to MSA- SlowFast. The addition of MPBHead raises mAP from the baseline of 70.58% to 75.09%. This improvement is mainly due to the module enhancing the model’s perception ability of spatial features at different scales, enabling it to effectively capture key features of large-scale global actions, such as tail-swaying, and small-scale local actions, such as head-shaking, simultaneously. The ST-CBAM module raises mAP to 74.32%, and its success lies in guiding the model to focus on the most discriminative segments and regions in the video sequence through attention mechanisms in both temporal and spatial dimensions. The BAF module achieves 72.16% mAP by promoting feature interaction and complementarity between the SlowFast dual-path.

The most outstanding performance stems from the joint application of MPBHead and ST-CBAM, which together elevated mAP by 7.05 percentage points over the baseline. This gain confirms the synergistic value of their complementary functions. Their core advantage lies in constructing an end-to-end optimization pipeline from feature capture to classification: the MPBHead ensures comprehensive extraction of multi-scale behavioral representations, particularly enhancing sensitivity to minority-class behaviors, while the ST-CBAM refines these features through spatio-temporal-channel attention, highlighting key discriminative information and suppressing background noise. This collaboration not only maintains the model’s sensitivity to diverse actions but also sharpens feature discriminability, enabling precise classification via a dynamically balanced decision mechanism.

Notably, the BAF module contributed a more limited improvement. This can be attributed to its functional overlap with the MPBHead. The MPBHead’s powerful multi-scale fusion already extracts and aggregates key spatial-temporal information, thereby diminishing the marginal benefit that the subsequent BAF module—designed for dual-path interaction—can provide. Consequently, when the MPBHead supplies high-quality fused features, the additional complementary effect of BAF becomes less pronounced.

## 4. Discussion

### 4.1. Interpretation of Research

Unlike current approaches that prioritize robustness through complex spatiotemporal architecture [38], and multimodal fusion [39,40], this study adopts a simplified, problem-centered strategy. Experimental results on our proprietary dataset demonstrate that the proposed MSA-SlowFast framework yields a clear performance gain over the classic SlowFast baseline for dairy cattle protective behavior detection, with an overall mAP improvement of 8.83 percentage points. This enhancement stems primarily from the synergistic effect of the three core modules integrated into our framework.

Firstly, MPBHead can generate more accurate classification results for different scales of behavior by adaptively integrating the features of different spatial semantic levels. This design idea is consistent with the general trend in the field of animal behavior recognition, that is, enhancing multi-scale feature representation is helpful for fine-grained action recognition. For example, Wang et al. [21] used an efficient 3D CNN in the recognition of basic motion behavior of dairy cows, and also stressed the importance of capturing the characteristics of different motion scales. However, unlike some methods based on pose estimation-based methods, this study directly processes the original video pixels, avoiding the risk that key point detection may fail in a complex pasture environment. Although this method, based on pixel-level spatio-temporal features, is more complex in calculation, it provides the possibility for direct detection of undefined local subtle movements of the body.

Secondly, ST-CBAM is introduced as an adaptive feature refinement mechanism. By sequentially applying channel attention and spatial attention, the model can suppress irrelevant background interference and dynamically focus on the spatio-temporal region that can best indicate protective behavior. This is particularly important for detecting fast and transient behaviors, because its key discrimination signal only exists in a small area and a short time window. In the field of animal husbandry, Liu et al. [22] added the attention mechanism to the improved SlowFast network to identify the sleeping and feeding behavior of horses, and also reported a significant improvement in performance, which is mutually confirmed with our findings and shows the universal effectiveness of the attention mechanism in the recognition of complex animal behavior.

Thirdly, BAF promotes more effective interaction between slow paths and fast paths in slow backbone networks. Different from simple splicing or summation, the BAF module learns to recalibrate and selectively fuse feature maps according to the text importance of feature maps, so as to produce a more robust unified spatio-temporal representation. This improves the discrimination of complex behaviors requiring joint understanding of posture and dynamics. Similar two-stream or two-branch fusion ideas have also been proven effective in other studies, Yuan et al. [23] used the serial feature cascade fusion of Res3D and BiLSTM in sheep behavior recognition. Our BAF module can be seen as a refinement of this idea. Its innovation lies in the realization of more refined and adaptive control of cross-channel information flow within the specific architecture of SlowFast, which is an important mechanism to improve the recognition ability of the model for complex cow protective behaviors (e.g., head-shaking with cow rotation).

Finally, the strategy for category imbalance is the direct reason for improving the detection of rare behavior. The timing-aware oversampling strategy and the adjustment of dynamic focus loss designed in this study directly address the severe challenge of the scarcity of protective behavior samples in datasets. The oversampling strategy increases the exposure of rare behavior samples in the training process by creating sample copies with time disturbance and enriches the learning experience of the model for a few categories. This refers to the oversampling idea of Chen et al. [33] for time series data and adapts it to video behavior clips. At the same time, the adopted focal loss forces the model to use more capacity to learn challenging, scarce protective behaviors by reducing the loss weight of the easily classified majority of normal behavior samples. Rezvani et al. [30] pointed out that similar loss function adjustment is one of the effective strategies in deep learning methods to solve class imbalance. Our experimental results show that the combined application of these strategies improves the recall rate of rare behavior categories without reducing the accuracy of most categories. This is consistent with the goal of Ni et al. [38] in dealing with data imbalance in multimodal cattle behavior classification, although they use different characteristic modes and network architectures.

### 4.2. Research Limitations and Future Directions

Unlike current approaches that prioritize robustness through complex spatiotemporal architecture [38], although the proposed MSA-SlowFast model has achieved promising results on our dataset, the findings also point to valuable directions for future research. On the one hand, like most data-driven methods, the performance of models is affected by the quality and diversity of training data. The proposed targeted oversampling strategy offers an efficient means of addressing class imbalance, yet its effectiveness could be further enhanced by a more precise characterization of protective behaviors in the initial samples. Additionally, given the temporal nature of video data, repeated sampling from a limited set of individuals carries the risk of learning environment-specific or cow-specific patterns rather than truly generalizable behavioral features. This limitation becomes particularly evident in complex farm settings, where occlusion or motion truncation often compromises data integrity, potentially constraining the model’s generalization to novel herds or unfamiliar environments. To build upon the current framework, future work could explore generative model-based augmentation techniques (e.g., using diffusion models [41]) as a complement to the proposed oversampling strategy. By synthesizing diverse yet authentic sequences of rare behaviors, such approaches could preserve essential action dynamics while varying non-essential attributes such as background, lighting, and individual appearance. This would not only mitigate the risk of overfitting but also enhance cross-environment robustness, ultimately extending the applicability of our method to a wider range of real-world scenarios. In particular, future work should prioritize validation on entirely held-out farms not seen during training, which would provide stronger evidence of generalization than the current train/val/test split from the same farms.

On the other hand, the experimental results clearly show that the model has significantly higher detection accuracy for large-scale global actions such as tail-swaying than for local subtle actions such as head-shaking and ear-flapping. This observation points to an area for further refinement: the current temporal sampling strategy, while effective for broader movements, could be optimized to better capture the rapid, fine-grained features characteristic of localized motions. The ST-CBAM module already strengthens spatio-temporal attention, and future work could build upon this foundation by adjusting frame sampling rates, designing denser temporal windows for localized actions, or incorporating complementary motion cues such as optical flow. These enhancements would further strengthen the model’s utility as a proactive welfare monitoring tool, enabling even earlier intervention capabilities.

Beyond these architectural and data-centric improvements, several practical considerations merit further investigation. First, real-time deployment constraints have not been explicitly addressed in the current study. Future work should establish latency benchmarks on edge devices commonly used in precision livestock farming (e.g., Jetson Orin [42] or Raspberry Pi-based systems) and explore model compression techniques such as pruning or knowledge distillation to enable on-farm real-time inference. Second, the current framework mostly looks at single-cow scenarios. However, on actual farms, protective behaviors from different cattle often overlap in both time and space, which creates ambiguous motion signals and occlusions. Extending the model to multi-cow scenes is essential for real-world deployment. Early efforts in this direction, such as the general video surveillance framework proposed by Zin et al. [43] for animal behavior analysis, have demonstrated the feasibility of multi-individual tracking, although often under controlled conditions. Instance-level tracking or spatio-temporal action localization represent promising technical pathways, as they enable the model to differentiate overlapping behaviors originating from different individuals. Third, to maximize the system’s utility as a proactive welfare monitoring tool, future work should aim for seamless integration with farm management systems as an end-to-end alert pipeline. This includes defining alert triggers based on confidence thresholds or temporal persistence of detected behaviors, designing user interfaces for farm staff, and validating the system under varying operational conditions. Such integration would enable even earlier intervention capabilities and transform the current proof-of-concept into a deployable decision-support tool.

In summary, although the MSA-SlowFast model performs well on the current dataset, moving toward robust and practical welfare monitoring in commercial farming will require further work on data diversity, fine-grained action recognition, real-time feasibility, multi-animal handling, and system integration.

## 5. Conclusions

The core contribution of this study is to propose a video detection method of dairy cattle protective behavior based on an improved SlowFast network. The experimental results show that the improved MSA-SlowFast model has achieved certain effectiveness on the self-built dairy cattle protective behavior dataset, with a mAP of 79.41%, which is 8.83% and 11.20% higher than the standard SlowFast model (70.58%) and Slow only model (68.21%), respectively. Further validation shows that the model exhibits high detection confidence in four specific actions labeled as protective behavior: 0.97 for tail swaying, 0.90 for head shaking, 0.92 for ear flapping, and 0.90 for leg kicking. Therefore, this study provides an effective method for the precise detection of instantaneous protective behavior in dairy cattle, which is of practical significance for individual health monitoring in intelligent animal husbandry. It offers a technical foundation for the intelligent and sustainable development of animal husbandry management.

## Figures and Tables

**Table 1:** Criteria for determining protective behavior.

Action	Judgment Criteria
tail-swaying	Swinging the tail left and right at different frequencies
head-shaking	Rapid swinging in the up and down or left and right directions, sometimes combined with head rotation movements.
ear-flapping	Quickly flap the ears in the forward and backward directions
leg-kicking	Rapid kicking of the front and rear legs towards the front or side

**Table 2:** The results of the comparison experiment.

Model	AP		mAP
	Normal Behavior	Protective Behavior	
SlowOnly (ResNet 50)	72.90	63.52	68.21
SlowOnly (ResNet 101)	77.75	61.13	69.44
SlowFast	77.73	63.43	70.58
MSA-SlowFast (ResNet 50)	79.96	72.46	76.21
MSA-SlowFast (ResNet 101)	82.54	76.28	79.41

**Table 3:** Experimental results of various loss functions in MSA-SlowFast.

Loss Functions	α	γ	W_y ^1^	mAP
BCE	-	-	-	69.30
Focal Loss	0.8	2.0	-	72.68
DFL	0.6	2.0	1.0:4.0	75.01
	0.6	3.0	1.0:4.0	75.83
	0.8	2.0	1.0:4.0	76.43
	0.8	3.0	1.0:4.0	76.10
	0.6	2.0	1.0:2.0	77.30
	0.6	3.0	1.0:2.0	78.09
	0.8	2.0	1.0:2.0	79.41
	0.8	3.0	1.0:2.0	78.17

^1^ represents the ratio of weight between normal behavior and protective behavior.

**Table 4:** Ablation study with MSA-SlowFast when MPBHead, ST-CBAM, and BAF are applied to the baseline.

SlowFast	MPBHead	ST-CBAM	BAF	mAP
√ **^1^**				70.58
√	√			75.09
√		√		74.32
√			√	72.16
√	√	√		77.63
√	√		√	74.77
√		√	√	75.52
√	√	√	√	79.41

^1^ ✓ denotes the use of the respective optimization module in the ablation study.

## Data Availability

The data that support the findings of this study are available from the corresponding author upon reasonable request.
